# Neighborhood Environments and Utilitarian Walking Among Older vs. Younger Rural Adults

**DOI:** 10.3389/fpubh.2021.634751

**Published:** 2021-06-04

**Authors:** Chanam Lee, Chunkeun Lee, Orion T. Stewart, Heather A. Carlos, Anna Adachi-Mejia, Ethan M. Berke, Mark P. Doescher

**Affiliations:** ^1^Department of Landscape Architecture and Urban Planning, Texas A&M University, College Station, TX, United States; ^2^California Department of Public Health, Sacramento, CA, United States; ^3^University of Washington, Seattle, WA, United States; ^4^Geisel School of Medicine at Dartmouth, Lebanon, NH, United States; ^5^Norris Cotton Cancer Center at Dartmouth-Hitchcock, Lebanon, NH, United States; ^6^Optum (United States), Eden Prairie, MN, United States; ^7^The Dartmouth Institute for Health Policy and Clinical Practice, Geisel School of Medicine at Dartmouth, Lebanon, NH, United States; ^8^University of Oklahoma Health Sciences Center, Oklahoma City, OK, United States

**Keywords:** physical activity, neighborhood environment, rural communities, older adults, walking

## Abstract

**Introduction:** Walking has the potential to promote health across the life span, but age-specific features of the neighborhood environment (NE), especially in rural communities, linked with walking have not been adequately characterized. This study examines the relationships between NE and utilitarian walking among older vs. younger adults living in US rural towns.

**Methods:** Data for this cross-sectional study came from telephone interviews in 2011–2012 with 2,140 randomly sampled younger (18–64 years, *n* = 1,398) and older (65+ years, *n* = 742) adults, collecting personal and NE perception variables. NE around each participant's home was also measured objectively using geographic information system techniques. Separate mixed-effects logistic regression models were estimated for the two age groups, predicting the odds of utilitarian walking at least once a week.

**Results:** Perceived presence of crosswalks and pedestrian signals was significantly related to utilitarian walking in both age groups. Among older adults, unattended dogs, lighting at night, and religious institutions were positively while steep slope was negatively associated with their walking. For younger adults, traffic speed (negative, –), public transportation (positive, +), malls (–), cultural/recreational destinations (+), schools (+), and resource production land uses such as farms and mines (–) were significant correlates of utilitarian walking.

**Conclusion:** Different characteristics of NE are associated with utilitarian walking among younger vs. older adults in US rural towns. Optimal modifications of NE to promote walking may need to reflect these age differences.

## Introduction

Walking, with all of its health benefits particularly for older adults, has the potential to promote health outcomes as adults age (1). According to the 2014 Behavioral Risk Factor Surveillance System (BRFSS) data, 26.9% of older adults aged 65–74 years reported no physical activity outside of work during the last month, and the number increased to 35.3% as age increased to 75 years and above (2). A growing body of research has begun to illuminate the differing roles that the neighborhood environment (NE) plays in promoting or, as is often the case, hindering walking as adults age (3).

Most studies to date about environment–walking relationships among older or general adults have been limited to metropolitan or urban areas. However, rural towns are home to 10% of the US population, have a disproportionately large number of older adults, and are aging more rapidly than the rest of the United States (4). Several recently published studies carried out in rural communities reported both similarities and differences in the correlates of walking between urban and rural residents (5–7). The study of Stewart et al. comparing one urban community and nine small rural towns in the United States found that the same land use (i.e., restaurants) can be positively associated with utilitarian walking in urban settings while negatively associated in rural settings, and NE is more strongly associated with utilitarian walking in urban communities. They also observed a higher prevalence of recreational walking in rural towns, and traffic speed was a significant predictor of recreational walking only in rural communities. The study of Doescher et al., further examining the same nine rural towns, reported that crosswalks, pedestrian signals, park/natural recreational areas, and manufacturing land uses were positively correlated with utilitarian walking in these communities (5). Another study in Japan explored the roles of street layout design and found that street intersection density was linked with increased walking for errands in urban areas, while street integration was positively associated with walking for commuting in rural areas (6). All of these studies were based on the general adult population. Little is known about the age-varying associations between walking and NE in these rural/small towns that are also known to be subject to significant health inequities (8).

Substantial empirical evidence based on general adult studies suggests that walkable neighborhoods typically feature compact development patterns, proximately located destinations, connected street/sidewalk networks, and safety from traffic and crime (9, 10). The roles of walkable neighborhood features, however, may differ among people from different age groups. One study indicated that NE may play a less significant role in walking for older adults compared to their younger counterparts (11). Another older adult study suggested that NE may be more important for influencing the amount of walking among those who already walk than encouraging non-walkers to walk (12). Age differences in transportation walking were found to be greater in lower walkability neighborhoods than in higher walkability neighborhoods (13). Other research pointed to the synergistic effects of NE and personal factors (e.g., socioeconomic status, self-efficacy, and personal barriers) on walking among older adults (14, 15). Studies examining self-report barriers and facilitators of walking and route choice models suggested that the characteristics of NE that influence walking behaviors in older adults might be highly fine-grained and location-specific (3, 16). One study found that the decline in walking for transport over a 4-year period was less among those living in walkable neighborhoods (17). A review study suggested that proximity to destinations, connected street networks, and safety from traffic were associated with older adults' mobility (18). Studies also showed that people with a longer residential history tend to be less fearful of their neighborhood (19) and walk more for exercise (20). These studies indicate that walkable NE has some potential to support mobility and aging in place.

The purpose of this study was to identify NE characteristics that are associated with utilitarian walking among older vs. younger adults living in US rural towns. This study focuses on utilitarian walking because it is associated more strongly with NE and less strongly with personal factors than recreational walking (7, 9) and because it is more likely to bring long-term lifestyle changes, is easily incorporated into the daily routine, helps preserve independent mobility of aging populations, and accompanies additional economic and environmental benefits resulting from reduced automobile use (21). Utilitarian walking is especially important for increasing or maintaining mobility and independence among older adults, supporting the aging in place initiatives (22, 23).

NE is the target setting of this study due to the increasing importance of the residential neighborhood among older adults, as they spend most of their time at home and rely more on proximately available resources within the neighborhood for their physical and psychosocial needs (24, 25). Furthermore, older adults tend to be more vulnerable to environmental challenges or barriers. For example, older adults walk at slower speeds and thus may have more difficulty crossing busy streets, especially when there are no crosswalks or when the crosswalk signals are too short (3).

The social ecological framework provides a theoretical foundation and guidance for this paper. It emphasizes the dynamic interplays between people and their environments (26). Compared to other common theories in the health promotion or behavior change literature that tend to focus on intrapersonal factors, the social ecological model draws attention to the social and physical environments as key determinants of individual health/behavioral outcomes (27). Lawton applies an ecological theory to describe the aging process as an evolving process of human adaptation to their environment (28). His theory highlights the importance of immediate contexts (social, physical, and technological) in determining such process and outcomes (28, 29). Both theories recognize the importance of environmental contexts such as neighborhoods in determining health behaviors such as walking and physical activity. They also agree that the nature of the environment–behavior relationships is highly dependent on the specific behavior, population, and the community context being targeted (30). These theories offer useful insights and support for environment–behavior studies to examine the population- and context-specific correlates of an explicit target outcome, such as utilitarian walking. Both theories further recognize the multilevel characteristics of the environment, including interpersonal, sociocultural, institutional, and physical environments; proximal to distal environments; and the interplay within and between factors at different levels (23). This study focuses on age (intrapersonal variable) and NE (both perceived and objectively measured environmental variables) to explore their roles in promoting or hindering utilitarian walking in US rural towns as the understudied settings for this type of study.

## Methods

This cross-sectional study examines the correlates of utilitarian walking in neighborhoods among 2,140 randomly sampled younger (18–64 years, *n* = 1,398) and older (65+ years, *n* = 742) adults. We used the age of 65 as the threshold in this study as it is the most commonly used and accepted threshold for defining older adults in the United States (e.g., Census Bureau, National Institutes of Health). Utilitarian walking in this paper is defined as walking to or from any destinations including recreational ones (e.g., grocery store, school, and park). Two separate mixed-effects multivariable logistic regression models were estimated for the two age groups, adjusting for the town-level clustering effect.

### Setting

In order to represent a diversity of rural towns in the United States, this study was carried out in nine towns from three diverse geographic regions: the Northwest (Washington), the Northeast (New Hampshire and New York), and the South (Texas) (Table 1). The selection criteria included: (a) geographically isolated rural towns located in counties classified as “micropolitan statistical areas” based on the US Census (31) with sufficient population (10,000–40,000) to support services for daily living; (b) clustered residential areas to permit walking between homes and routine destinations; (c) diverse racial/ethnic composition and education/income levels; and (d) availability of geographic information systems (GIS) data. More information about the study setting and data collection methods can be found elsewhere (Blinded for Review, 2016).

**Table 1:** Survey respondents by town and by age group.

**Region**	**City, state**	**Size (mi.^**2**^)^a^**	**Population^a^**	**Density^a^^,^^b^**	**Income (US $)^a^^,^^c^**	**Younger adults (18–64 years)**		**Older adults (>65 years)**	
						**Freq**.	**%**	**Freq**.	**%**
Northwest	Walla Walla, WA	10.82	31,731	2,933	41,236	173	77.6	50	22.4
	Moses Lake, WA	10.18	20,366	2,001	47,535	148	66.1	76	33.9
	Aberdeen, WA	10.62	16,896	1,591	39,530	166	68.0	78	32.0
Northeast	Plattsburgh, NY	5.04	19,989	3,966	35,528	145	66.2	74	33.8
	Berlin, NH	61.70	10,051	163	38,107	144	66.7	72	33.3
	Lebanon, NH	40.36	13,151	326	54,969	223	73.8	79	26.2
South	Kerrville, TX	16.70	22,347	1,338	41,064	99	40.7	144	59.3
	Huntsville, TX	30.90	38,548	1,248	29,465	138	58.2	99	41.8
	Bay City, TX	8.49	17,614	2,075	37,601	162	69.8	70	30.2
Total						1,398	65.3	742	34.7

a *Census 2010*.

b *Persons/square mile*.

c *Median household income*.

### Survey

All personal variables were obtained from an ~20-min-long telephone survey administered in 2011 in both English and Spanish. The survey instrument was developed by taking items used in previous peer-reviewed research, including the International Physical Activity Questionnaire (32), the Neighborhood Environment Walkability Scale (33), the Behavioral Risk Factor Surveillance System (34), and the Rural Active Living Perceived Environment Support Scale (35), and by pilot testing it with 50 randomly sampled respondents from the same study population. The final survey instrument included demographics, race and ethnicity, health and socioeconomic status, barriers and facilitators of walking, walking and sedentary behaviors, and neighborhood perceptions. The survey protocol and instrument were approved at each of the investigators' universities.

The study used a spatial sampling strategy that involved random sampling of residential units from those selected into the sample frame (36). The sample frame was spatially delineated to include all census blocks that contained the top 80% of the population in each study town. This excluded very low-density residential areas often located in farmland and undeveloped areas wherein no nearby destinations were available for utilitarian walking. Phone numbers were identified through a reverse directory landline lookup for the selected units, which yielded an approximate matching rate of 40%. The phone interviews were conducted over four months in 2011 when the weather was most favorable for walking in each region, with a maximum of nine callbacks and an estimated response rate of 18.8%. The respondent eligibility criteria were (a) aged 18 years or older; (b) resided at the current address for at least 1 year; and (c) being able to walk without special equipment for 5 minutes.

### Geographic Information Systems

GIS was used to generate the objective measures of NE related to walkability. Raw data were obtained from each jurisdiction, and additional data were collected from aerial photos, online maps, and various agencies (e.g., tax assessor's office, parks and recreation department, and transit agency). A detailed protocol and definition for each GIS measure was developed and followed to ensure valid and consistent measures across all nine towns. GIS measurements were carried out in 2013 and included buffer-based measures (e.g., total number of banks and average residential unit density), which were taken from a 1-km street network “sausage” buffer (37) around each survey respondent's home, and proximity measures (e.g., distance to the closest park) taken as the shortest distance from the home to each target destination along the road network up to 2 km. The sausage buffer method is similar to the standard street network buffer, but it excludes interior areas inside the street block where pedestrians are not likely to see or get to. Details about the GIS method, measures, and protocols used in this study can be found in a previously published paper (Blinded for Review, 2016).

### Variables

The outcome was a binary variable, walking at least once a week vs. not. It was generated from the survey questions asking about the number of times per month walked from home to a series of common destinations, which were converted to weekly frequencies. Predictor variables included personal and environmental variables. Descriptive statistics and the coding schemes of only those that retained statistical significance at the 0.1 level in the final multivariable models are included in Tables 2 and 3. All personal variables were derived from the survey data and consisted of five domains: demographics, health and socioeconomic status, behavior, barrier to walking, and residential self-selection. The environmental variables came from both survey (neighborhood perception) and GIS (objective built environment). Neighborhood perception variables (11 variables considered) included safety, street/traffic conditions, visual quality, sidewalk availability, shade condition, and presence of destinations. The GIS variables included eight sub-domains: generalized land use (19 variables), destination land use (102), residential density (six), transportation infrastructure (52), economic environment (nine), employment (six), regional location (two), and natural environment (six).

**Table 2:** Descriptive statistics and bivariate tests of the study variables used in the final models: personal variables (survey).

**Domain**	**Variable**	**Younger adults**		**Older adults**	
		**Freq. or mean**	**% or SD**	**Freq. or mean**	**% or SD**
Demographics	Total *N*	1,398		742	
	Age (years)	48.8	11.1	74.1	6.7
	*Gender*: χ^2^ = 0.385, *p* = 0.535				
	Male (ref.)	531	38.0	292	39.4
	Female	867	62.0	450	60.6
Health and socioeconomic status	*Household annual income* (US $): χ^2^ = 85.819, *p* < 0.000 | *t* = 7.151, *p* < 0.001				
	≤ 25,000	237	19.1	155	25.1
	25,001–50,000	261	21	221	35.8
	50,001–100,000	509	41	194	31.4
	>100,000	233	18.8	47	7.6
	Nine-category version (1: lowest−9: highest)^a^	5.6	2.1	4.9	1.9
	*Education*: χ^2^ = 19.931, *p* = 0.001 | *t* = −1.446, *p* = 0.148				
	≤ High school graduate	389	27.9	215	29.1
	Some college/associate degree	400	28.6	197	26.6
	≥College graduate	608	43.5	330	44.5
	Seven-category version (1: lowest−7: highest)^a^	5.2	1.3	5.3	1.3
	*Difficulty walking*: χ^2^ = 46.535, *p* < 0.000				
	Not at all difficult (ref.)	1,321	94.5	637	85.9
	Difficult or do not do this activity	77	5.5	105	14.1
Behavior	*Utilitarian walking* (h/week): χ^2^ = 80.237, *p* < 0.001				
	Non-walker (ref.)	287	20.5	286	38.5
	Walker	1,111	79.5	456	61.5
	*Recreational walking* (h/week): χ^2^ = 22.470, *p* = 0.001 | *t* = 3.114, *p* = 0.002				
	0 [0]	110	7.9	101	13.6
	0.1–0.5 [1]	166	11.9	100	13.5
	0.6–1.5 [2]	273	19.5	121	16.3
	1.6–2.5 [3]	205	14.7	113	15.2
	2.6–5.0 [4]	312	22.3	150	20.2
	5.1–7.0 [5]	119	8.5	56	7.6
	7.1+ [6]	213	15.2	101	13.6
	Seven-category version (0: lowest - 6: highest)*^a^*	3.2	1.8	2.9	1.9
	*Screen time* (h/week): *t* = −5.815, *p* < 0.001	15.6	12.9	19.2	14.5
Walking barrier	*Lack of time* (Does this keep you from walking?): χ^2^ = 215.839, *p* < 0.001				
	Yes	782	56.1	169	22.9
	No (ref.)	612	43.9	570	77.1
Residential self-selection	*Ease of walking to retail, services, and transit* (Was this important in choosing where to live?): χ^2^ = 11.096, *p* = 0.001				
	Yes	523	37.7	223	30.5
	No (ref.)	863	62.3	509	69.5

*Coding for income (in US $): 1: ≤ 10,000; 2: 10,001–15,000; 3: 15,001–25,000; 4: 35,001–35,000; 5: 35,001–50,000; 6: 50,001–75,000; 7: 75,001–100,000; 8: 100,001–150,000; 9: ≥150,001. Coding for education: 1: Never attended school; 2: Elementary; 3: Some high school; 4: High school graduate; 5: Some college/associate degree; 6: College graduate; 7: Graduate school or more*.

a *Originally captured as ordinal categorical variables and treated as continuous variables in the multivariable models*.

**Table 3:** Descriptive statistics and bivariate tests of the study variables used in the final models: environmental variables (survey and GIS).

**Domain**	**Variable**	**Younger adults**		**Older adults**	
		**Freq. or mean**	**% or SD**	**Freq. or mean**	**% or SD**
Neighborhood perception (survey)	*Crosswalks and pedestrian signals* (There are crosswalks and pedestrian signals to help walkers cross busy streets in my neighborhood): χ^2^ = 21.281, *p* < 0.001				
	Agree	810	58.2	350	47.8
	Disagree (ref.)	581	41.8	383	52.2
	*Sidewalks or shoulders* (There are sidewalks or shoulders where people can walk in my neighborhood): χ^2^ = 5.000, *p* = 0.025				
	Agree	1,027	74.1	509	69.5
	Disagree (ref.)	359	25.9	223	30.5
	*Unattended dogs* (Unattended dogs are a problem in my neighborhood): χ^2^ = 14.182, *p* < 0.001				
	Agree	245	17.6	84	11.4
	Disagree (ref.)	1,149	82.4	654	88.6
	*Well lit at night* (My neighborhood is well lit at night): χ^2^ = 6.618, *p* = 0.010				
	Agree	865	62.8	490	68.4
	Disagree (ref.)	513	37.2	226	31.6
	*Slow traffic speed* (The speed of traffic on most nearby streets is usually slow): χ^2^ = 0.0049, *p* = 0.944				
	Agree	1,031	75.0	543	74.9
	Disagree	343	25.0	182	25.1
Generalized land use (GIS)	*Resource production and extraction land uses* (% area within buffer): χ^2^ = 2.614, *p* = 0.271				
	0% (ref.)	660	47.5	321	44.0
	0.1–3.0%	418	30.1	240	32.9
	>3.0%	311	22.4	169	23.1
	*Cultural, entertainment and recreational land uses* (% area within buffer): χ^2^ = 18.870, *p* < 0.001				
	0% (ref.)	285	20.5	149	20.4
	0.1–1.5%	404	29.1	235	32.2
	1.6–4.0%	460	33.1	182	24.9
	>4.0%	240	17.3	164	22.5
Destination land use (GIS)	*Religious institutions* (presence within buffer): χ^2^ = 10.207, *p* = 0.001				
	Absence (ref.)	1,091	78.0	533	71.8
	Presence	307	22.0	209	28.2
	*Schools* (total counts within buffer): *t* = 4.585, *p* < 0.001	1.482	1.3	1.203	1.3
	*Malls* (presence within buffer): χ^2^ = 1.488, *p* = 0.222				
	Absence (ref.)	1,139	82.0	614	84.1
	Presence	250	18.0	116	15.9
Transportation (GIS)	*Public transportation* (total counts within buffer): χ^2^ = 0.5934, *p* = 0.441				
	Absence (ref.)	1,279	92.1	679	93.0
	Presence	110	7.9	51	7.0
Natural environment (GIS)	*Slope* (mean % within buffer): χ^2^ = 0.307, *p* = 0.580				
	≤ 8.33% (ref.)	1,344	96.8	703	96.3
	>8.33%	45	3.2	27	3.7

### Statistical Analysis

We used mixed-effects multivariable logistic regression models to account for the town-level data clustering and identified the factors significantly associated with the odds of walking at least once a week in the neighborhood to reach a destination. The interclass correlation coefficient (ICC) values of the final multivariable models were 0.048 in the older adult model and 0.011 in the younger adult model. This means that the town-level effect accounts for 4.8% and 1.1% of the total variances explained in the older and younger adult models, respectively.

Due to the lack/shortage of theoretical foundations to guide the selection of the environmental variables, especially the GIS variables that tend to be highly correlated with each other, a three-step modeling process was employed to systematically test and isolate the most significant variables: (1) estimation of the base model with the personal variables only; (2) one-by-one test of the environmental variables by adding one environmental variable at a time to the base model; and (3) estimation of the final model by considering all the significant variables identified in step 2. To further examine the moderation effect of the age variable, we carried out a formal moderator test (38). It involved adding the interaction terms between age and the predictor variables, one at a time, to the final model. The statistical significance was set to *p* < 0.10 in steps 1 and 2 for more thorough considerations of all potential predictors and given the data-driven nature of the screening process (step 2) that was necessary, although not ideal, for the environmental variables. In the final models, we stayed with the standard alpha level of 0.05 for reporting and discussing the significant findings. All statistical analyses were carried out in 2013 using STATA version 12.0 (StataCorp LP, College Station, TX).

## Results

The participants (1,398 younger and 742 older adults) were primarily white and non-Hispanic, with 80.0% of the younger adults and 91.5% of the older adults being white and 85.5% and 97.0%, respectively, reporting a non-Hispanic origin. Based on the body mass index estimated from the self-reported weight and height, 26.1% of the younger adults belonged to the obese category compared to only 17.5% among the older adults. About 4.5% of the older adults, compared to 3.7% among the younger adults, lived in a household without a car; 57.1% of the older and 72.8% of the younger adults were married or lived with a partner. Table 4 displays the significant correlates of walking identified for each age group after adjusting for other significant covariates.

**Table 4:** Multilevel correlates of neighborhood utilitarian walking among younger vs. older adults: results from multivariable mixed-effects models.

**Domain**	**Variable^†^**	**Older adults**				**Younger adults**			
		**Odds ratio**	***p*-value**	**95% CI**		**Odds ratio**	***p*-value**	**95% CI**	
				**Lower**	**Upper**			**Lower**	**Upper**
**Personal correlates (survey)**									
Demographics	Female	0.513^**^	**0.003**	0.329	0.799	0.527^**^	** <0.001**	0.374	0.742
	Age (years)					0.974^**^	**0.001**	0.959	0.989
Health and socioeconomic status	Education (seven ordinal categories)	1.332^**^	**0.004**	1.094	1.623				
	Income (nine ordinal categories)	0.850^*^	**0.026**	0.737	0.981	0.920	0.057	0.844	1.002
	Difficulty in walking	0.273^**^	** <0.001**	0.150	0.496				
Behavior	Recreational walking (seven ordinal categories)	1.342^**^	** <0.001**	1.196	1.506	1.467^**^	** <0.001**	1.330	1.617
	Screen time (h/week)	0.978^**^	**0.004**	0.963	0.993				
Walking barrier	Lack of time	2.254^**^	**0.002**	1.355	3.747				
Residential self-selection	Ease of walking to retail, services, and transit	1.735^*^	**0.033**	1.044	2.884				
**Environmental correlates—neighborhood perception (survey)**									
Neighborhood perception	Unattended dogs	3.071^**^	**0.002**	1.532	6.158				
	Well lit at night	1.648^*^	**0.029**	1.052	2.584				
	Crosswalks and pedestrian signals	1.806^*^	**0.012**	1.139	2.863	1.713^**^	**0.002**	1.224	2.397
	Sidewalks or shoulders	1.486	0.098	0.929	2.377				
	Slow traffic speed					1.537^*^	**0.016**	1.084	2.179
**Environmental correlates—objective built environment (GIS)**									
Generalized land use	Resource production and extraction land uses (% area within buffer)								
	0.1–3.0% (ref.: 0%)					0.590^*^	**0.010**	0.394	0.882
	>3% (ref.: 0%)					0.355^**^	** <0.001**	0.229	0.551
	Cultural, entertainment, and recreational land uses (% area within buffer)								
	0.1–1.5% (ref.: 0%)					1.538	0.058	0.985	2.402
	1.6–4.0% (ref.: 0%)					2.058^**^	**0.004**	1.264	3.352
	>4.1% (ref.: 0%)					1.589	0.083	0.941	2.683
Destination land use	Religious institutions (presence within buffer)	1.920^**^	**0.009**	1.176	3.134				
	Schools (counts within buffer)					1.224^**^	**0.007**	1.056	1.418
	Malls (presence within buffer)					0.601^*^	**0.022**	0.388	0.931
Transportation	Public transportation (presence within buffer)					3.498^*^	**0.011**	1.330	9.198
Natural environment	Slope (mean % slope within buffer: >8.33% or >1:12 slope, ref: ≤ 8.33%)	0.334^*^	**0.049**	0.112	0.995				

*Older adults model: N = 548, pseudo-R^2^ = 0.226, AIC = 589.118, BIC = 653.713*.

*Younger adults model: N = 1,207, pseudo-R^2^ = 0.196, AIC = 1,000.963, BIC = 077.402*.

† *See Tables 2, 3 for detailed variable coding schemes*.

*Boldface indicates statistical significance (*

* *p < 0.05*,

** *p < 0.01)*.

### Personal Correlates

From the multivariable analyses, we found two personal variables associated with home-based utilitarian walking regardless of age. Females were less likely to walk to destinations than males in both age groups, with odds ratios (ORs) of 0.53 (*p* < 0.001) for the younger adults and 0.51 (*p* = 0.003) for the older adults. Those who walked for utilitarian purposes also walked more for recreational purposes regardless of their age group (*p* < 0.001 in both models).

Age was negatively associated with walking in the younger adult model only (OR = 0.98, *p* = 0.001). A 1-year increase in age was associated with an ~2.5% decrease in the odds of walking for utilitarian purposes. On the other hand, education (OR = 1.33, *p* = 0.004) and time barrier (OR = 2.25, *p* = 0.002) were positively while income (OR = 0.85 and *p* = 0.026), difficulty in walking (OR = 0.27, *p* < 0.001), and screen time (OR = 0.98, *p* = 0.004) were negatively associated with walking among the older adults only. The ease of walking to retail, services, and transit being considered when choosing where to reside served as a proxy for residential self-selection and showed a positive relationship with walking in the older adults only.

### Environmental Correlates

One environmental variable was significant in predicting the odds of home-based utilitarian walking in both age groups: perceived presence of crosswalks and pedestrian signals (OR = 1.81, *p* = 0.012, for the older adults; OR = 1.71, *p* = 0.002, for the younger adults).

For the older adults, perceptions related to having more unattended dogs (OR = 3.07, *p* = 0.002) and better lighting conditions (OR = 1.65, *p* = 0.029) were positively associated with utilitarian walking. The presence of religious institutions within the 1-km home buffer (OR = 1.92, *p* = 0.009) was positively while more sloped (>8.33% or >1:12 slope) areas within the buffer (OR = 0.33, *p* = 0.049) were negatively associated with walking among the older adults.

For the younger adults, perceptions of slow traffic speed in the neighborhood were positively associated with walking (OR = 1.54, *p* = 0.016). From the objective variables, the amounts of cultural–entertainment–recreational land use (e.g., public parks, private resorts, and places of assembly) were positively while resource production and extraction land uses (e.g., farms and mines) were negatively associated with utilitarian walking. Objective measured availability of public transportation captured as the presence of intercity transit stops (OR = 3.50, *p* = 0.011) and the presence of schools (OR = 1.22, *p* = 0.007) within the 1-km buffer from home were positive predictors; the presence of malls (OR = 0.60, *p* = 0.022) within the buffer was a negative predictor of walking among the younger adults.

### Moderator Test of Age Effects

**Figure 1:**
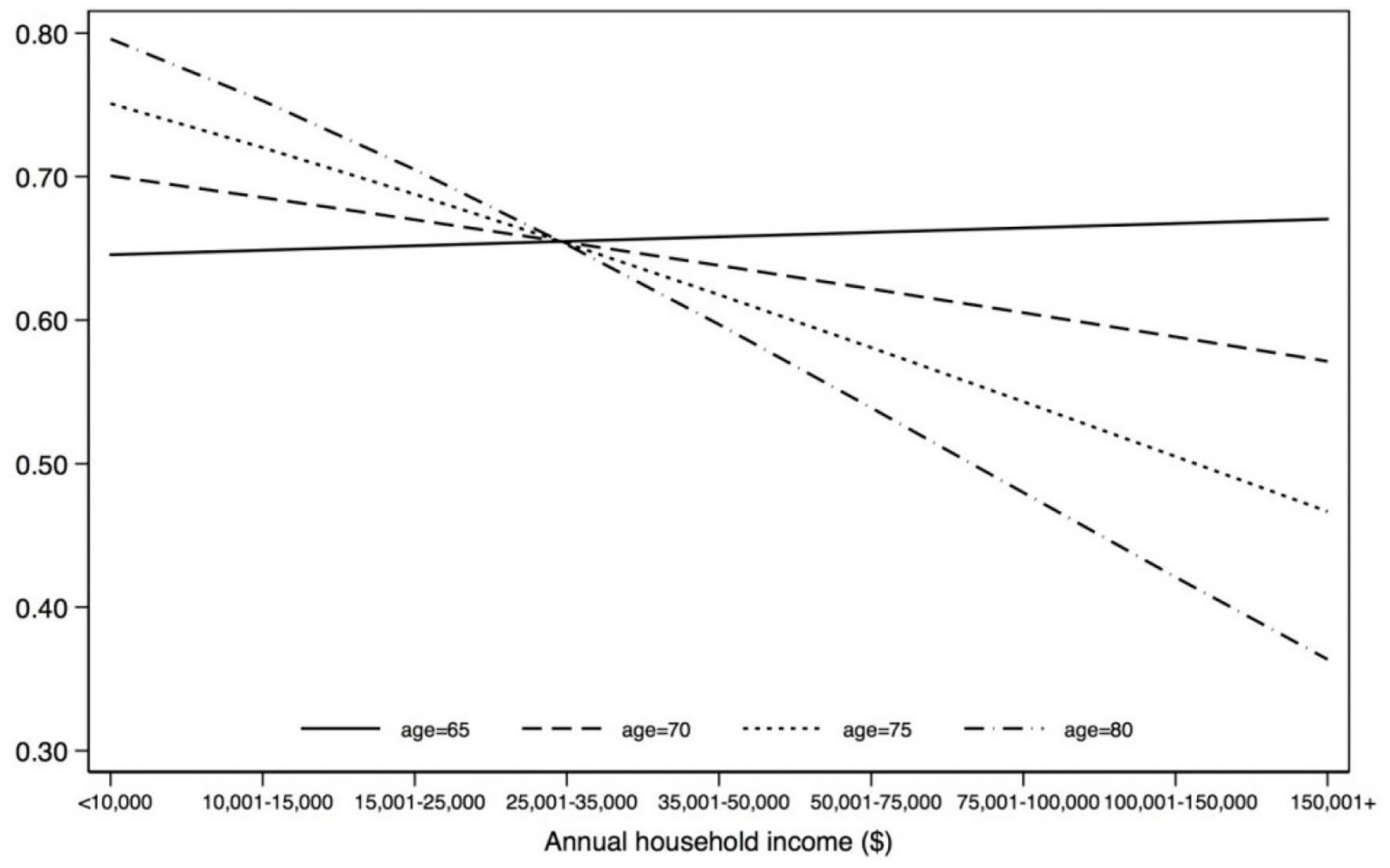
Moderating effect of age: predicted probability of becoming a utilitarian walker by age and income (older adult model). Age (OR = 1.096, *p* = 0.046, 95% CI = 1.002–1.200); income (OR = 4.623, *p* = 0.019, 95% CI = 1.286–16.613); age*income (OR = 0.977, *p* = 0.008, 95% CI = 0.960–0.994).

**Figure 2:**
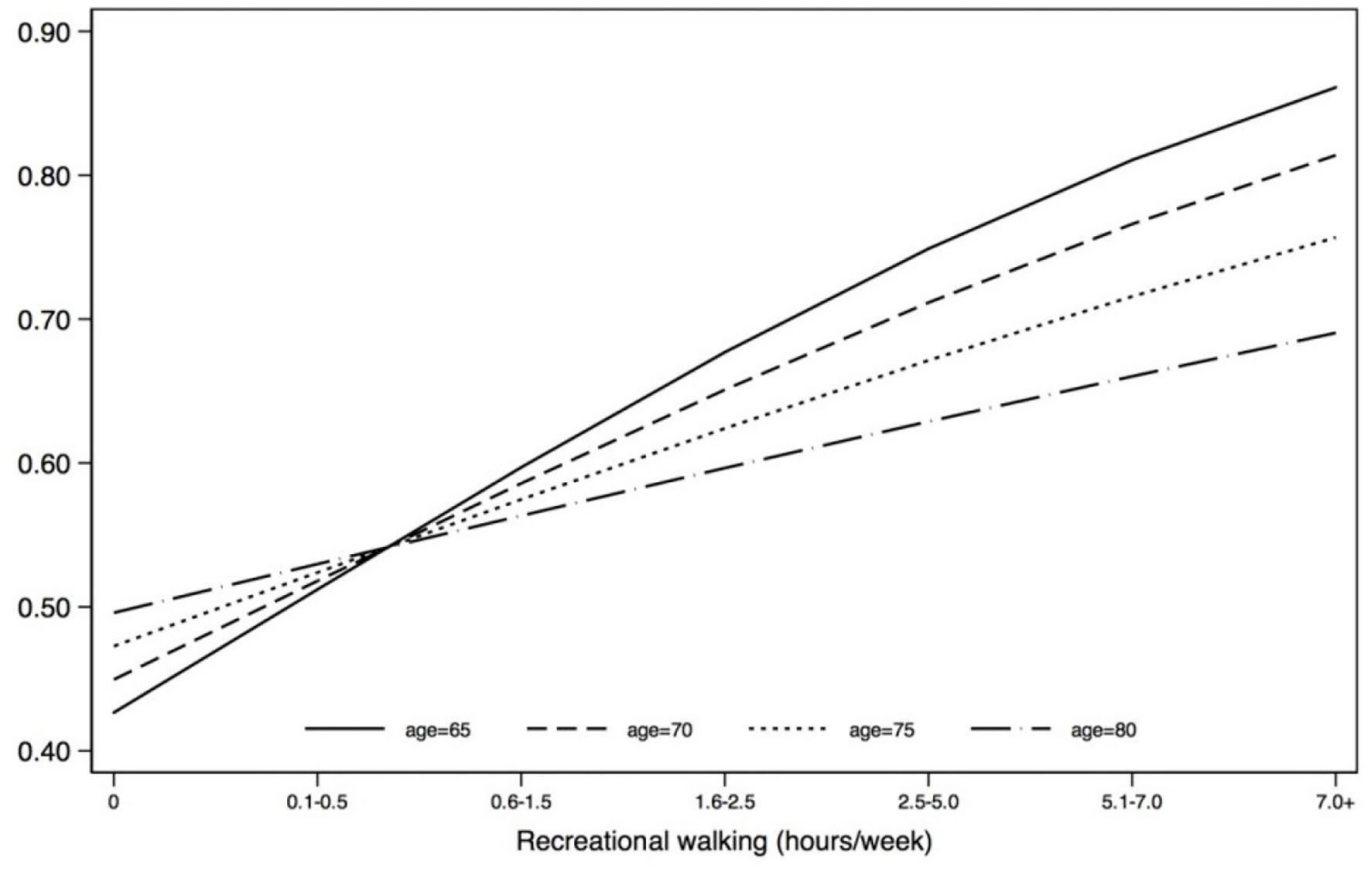
Moderating effect of age: predicted probability of becoming a utilitarian walker by age and recreational walking (older adult model). Age (OR = 1.025, *p* = 0.360, 95% CI = 0.972–1.080); recreational walking (OR = 1.348, *p* < 0.001, 95% CI = 1.197–1.518); age*recreational walking (OR = 0.982, *p* = 0.029, 95% CI = 0.966–0.998).

No significant interaction terms were found for the younger age model. For the older adults, two interaction terms were significant: age^*^income and age^*^recreational walking. Figures 1, 2 show the predicted probability of becoming a utilitarian walker across the different age ranges (within the older adult group) by income and by recreational walking. The results indicate that age intensifies the negative relationship between annual household income and the probability of utilitarian walking, while age attenuates the positive relationship between hours of recreational walking and the probability of utilitarian walking.

## Discussions and Conclusion

### Older Adults

We found that both personal and environmental characteristics were associated with adults' utilitarian walking in rural US towns. The findings suggest that walkable NE for older adults could focus on safety-related features. All four significant environmental variables, including lighting, unattended dogs, crosswalks/pedestrian signals, and slope, were directly or indirectly related to the multifaceted concept of safety (39). In addition, perceived availability of sidewalks/shoulders (OR = 1.49, *p* = 0.098) which approached the significant level is relevant to pedestrian safety. This finding is consistent with previous studies reporting environmental factors associated with utilitarian walking in neighborhoods, in which safety has been one of the most frequently documented domains of correlates (9, 40, 41). Furthermore, older adults are more vulnerable to safety-related environmental challenges due to their functional and cognitive declines (28), and therefore providing safe and barrier-free environments may hold even greater importance to support their walking. One specific finding on unattended dogs that had a positive relationship with walking may be considered counterintuitive, but this can be attributable to the likelihood that those who walk more are more likely to observe unattended dogs. The findings on unattended dogs from previous studies have been inconsistent. For example, a study on the correlates of physical activity among African American women in South Carolina found no significant relations between stray dogs and physical activity (42), while another study among middle-aged and older women reported positive relationships between unattended dogs and physical activity (43).

Steep slope was negatively associated with older adults' utilitarian walking in this study, which was defined as >8.33% (1:12 slope), the maximum slope allowed for wheelchair ramps (44). Previous studies on recreational or exercise walking have reported positive roles of slope among older adults (45) and among adults in general (46). The positive relationships with recreational/exercise walking could potentially be due to hilly areas' co-occurring features and benefits such as attractive views and increased exercise benefits, while hilly terrains may function as a barrier to utilitarian walking in which the walker is primarily interested in reaching the destination easily.

Among the land use-related GIS variables examined in this study, only one variable, having one or more religious institutions within 1 km from their home, was shown to be positively associated with walking among the older adults. This finding suggests limited roles of the land use domain for older adults' walking while also suggesting the strong potential for religious institutions to serve as multifunctional destinations not only for religious services but also for other sociocultural and service activities among older adults. The health beneficial roles of religious involvement have been previously reported, including mortality, well-being, and social support (47, 48). This study's finding on the role of religious institutions as walking-friendly destinations suggests that these institutions may also serve to bring additional health benefits to community-dwelling older adults.

The moderator test for the age effect revealed that two age interaction terms were significant. The age and recreational interaction effect suggests that the positive relationship between hours of recreational walking and the probability of utilitarian walking was weakened with older age. This finding also implies that the two different purposes of walking, recreational and utilitarian, in our study are mutually reinforcing (rather than replacing), but its magnitude is attenuated with age. Income showed an opposite pattern of association. For example, at the age of 65, income is estimated to have little impact on the probably of utilitarian walking. At the age of 80, income is expected to have a strong negative association with the probably of walking (ranging from 0.80 of walking probability for those with less than US $10,000 per year of household income to about 0.35 among those earning more than US $150,000).

### Younger Adults

More environmental factors, compared to the personal factors and to older adults, were found to be significant for younger adults' walking. From the neighborhood perception domain, perceptions of slow traffic speed and presence of crosswalks and pedestrian signals in the neighborhood were positively associated with their walking. Perceived presence of crosswalks and pedestrian signals was the only environmental variable that showed significance in both age groups and, therefore, worth attention as an intervention target given its consistent significance and its relative affordability for installation. We would anticipate effective interventions from combining crosswalks with raised traffic tables to reduce the traffic speed and/or with pedestrian signals to further enhance pedestrian safety. In addition, recreational walking showed a positive relationship with utilitarian walking in both age groups. Specific relationships (reinforcing, substituting, etc.) between the different types/purposes of walking have not been fully explored in previous studies, and this study adds helpful insights on their relationships.

Land uses were important for the younger adults' walking, more so than for the older adults. The results suggested that incorporating cultural, entertainment, and recreational land uses (e.g., public parks, private resorts, and places of assembly) and schools with walking/running tracks and other recreational facilities open to the public into residential communities could facilitate younger adults' utilitarian walking. However, resource production/extraction land uses (e.g., farms and mines) and malls, which tend to occupy large land areas with extensive surface parking and limited pedestrian accessibility, could discourage their walking. This finding is consistent with previous studies that reported generally positive roles of destinations, measured as land use mix, accessibility to places, etc., in promoting adults' utilitarian walking (9, 49).

### Limitations

The findings from this study may not be generalizable to areas other than the study towns. However, this study went beyond most previous studies that were carried out in a single community by including nine towns from three diverse regions. As a cross-sectional study, only correlational associations among the variables can be established, and there are likely missing covariates not captured in our study, such as additional correlates that may be important to one or the other age group only. The response rate could have been higher with different or additional survey methods, but not feasible for this multi-year, multi-region study. Our respondents had lower representations of younger, male, and Latino populations when compared to the Census data (Blinded for Review, 2014), possibly due to its participant recruitment through landline phone numbers. No formal reliability or validity test results are available for our survey instrument. However, most survey items were adopted directly or modified from existing surveys, and the instrument was finalized after a series of pilot testing. While we considered residential preferences and attitudinal factors related to walking, it is still possible that respondents, compared to non-respondents, comprised those who were more likely to walk or over-reported their walking. However, we do not believe that such possibilities vary by the NE characteristics, which are the key independent variables in this study (Blinded for Review, 2014). We found that perceived lack of time was associated with higher amounts of walking among the older adults. We were not able to use these data to further explore this puzzling finding, but it is possible that those who engage in walking are more likely to be aware of or sensitive to barriers to walking, such as a lack of time.

### Conclusion

Our findings suggest that, in rural US towns, NE influences home-based utilitarian walking for all adults. However, safety and slope are important primarily for the older adults, while the availability of recreational opportunities and the absence of malls have more prominent roles for the younger adults. For the older adults, relatively low-cost NE features such as crosswalks and lighting appear effective in stimulating their walking, making them appealing intervention targets especially given the growing number of older adults in the United States. For younger adults, additional interventions requiring longer-term land use changes appear necessary. Increased attention to NE by policymakers and professionals in aging, public health, transportation, and urban planning sectors could lead to increased walking among older and younger adults in rural towns.

## Data Availability Statement

The raw data supporting the conclusions of this article will be made available by the authors, without undue reservation.

## Ethics Statement

This study was reviewed and approved by the Institutional Review Boards at the three participating institutions: Texas A&M University, University of Washington, and Dartmouth College.

## Author Contributions

ChaL: conceptualization, research design, data analysis and interpretation, manuscript writing, and review and finalization. ChuL: data analysis, data interpretation, and manuscript writing. OS: conceptualization, research design, manuscript writing, and data interpretation. HC: conceptualization, data collection, and manuscript review. AA-M: research design, data interpretation, and manuscript review. EB: conceptualization, data interpretation, and manuscript review. MD: research design, conceptualization, data interpretation, and manuscript review. All authors contributed to the article and approved the submitted version.

## Conflict of Interest

The authors declare that the research was conducted in the absence of any commercial or financial relationships that could be construed as a potential conflict of interest.
